# Long-term clinical outcomes of endoscopic retrograde cholangiopancreatography for pancreatic duct stone treatment in children with chronic pancreatitis

**DOI:** 10.1371/journal.pone.0336638

**Published:** 2026-02-27

**Authors:** Yong Lv, Fangdi Yu, Bo Xiang

**Affiliations:** 1 Department of Pediatric Surgery, West China Hospital, Sichuan University, Chengdu, China; 2 Department of Endocrinology and Metabolism, West China Hospital, Sichuan University, Chengdu, China; 3 West China School of Nursing, Sichuan University, Chengdu, China; Faculty of Medicine Vajira Hospital, Navamindradhiraj University, THAILAND

## Abstract

**Background:**

Evidence on long-term outcomes of endoscopic retrograde cholangiopancreatography (ERCP) for pancreatic duct stones (PDS) in children with chronic pancreatitis (CP) remains scarce. This study aimed to evaluate the long-term clinical outcomes of ERCP in this population.

**Methods:**

A single-center retrospective cohort study was conducted on children with CP and PDS who underwent ERCP at our Hospital (2008.1.1–2024.12.31). Follow-up assessed stone clearance, complications, pain relief, pain type, growth, quality of life (QoL), and long-term complications.

**Results:**

A total of 90 children with CP and PDS were enrolled in this single-center retrospective cohort study. ERCP achieved 72.1% complete stone clearance; overall complications were 9.6%. Among the 90 patients, 77 (85.6%) completed the full follow-up. Post-ERCP, all abdominal pain subtypes significantly decreased (P < 0.001), with 70.1% complete pain relief. Height and weight increased (P < 0.01); QoL (PedsQL™4.0) scores rose from 69 to 85 points (P < 0.001). Long-term complications included new diabetes (5.2%) and steatorrhea (5.2%); PDS recurrence was 7.8%.

**Conclusion:**

ERCP is safe and effective for children with CP and PDS, significantly improving long-term pain, growth, and QoL, supporting its role as a first-line therapy.

## 1. Introduction

Chronic pancreatitis (CP) is a chronic disease characterized by irreversible changes in pancreatic structure and insufficiency of pancreatic secretion functions, caused by multiple factors. With its prolonged disease course and complex complications, CP has become one of the diseases threatening the digestive system health and growth of children [1]. Unlike adult CP, which is mainly caused by alcohol, the etiology of pediatric CP has distinct characteristics—hereditary pancreatitis, pancreaticobiliary anatomical abnormalities, and sequelae of recurrent acute pancreatitis are the main pathogenic factors [2]. Pancreatic duct stones (PDS) are the most common pathological change in patients with CP. Their formation not only leads to pancreatic duct obstruction but also induces recurrent abdominal pain. In severe cases, it even affects the growth of children and reduces their quality of life [3].

Currently, the core goals of treating CP combined with PDS in children are “relieving pancreatic duct obstruction, alleviating symptoms, and protecting pancreatic function”. Treatment options include endoscopic therapy, surgical operation, and extracorporeal shock wave lithotripsy [4,5]. Among them, endoscopic retrograde cholangiopancreatography (ERCP) has become a first-line intervention due to its minimally invasive advantage, which enables direct clearance or drainage of pancreatic duct stones [6]. However, the physiological particularities of children pose unique challenges to the application of ERCP. The success rate of ERCP intubation in children with a body weight <10 kg is significantly reduced, and the failure rate is positively correlated with low body weight. The incidence of post-ERCP pancreatitis (PEP) ranges from 14.5% to 20.7% [7–9]. Although existing studies have initially verified the short-term efficacy of ERCP in the treatment of pediatric CP, there remains a significant gap in evidence regarding the long-term outcomes of PDS [10]. Published long-term studies have limitations: either the sample size is extremely small, or they do not focus on the PDS population, only evaluating the overall symptom improvement of CP, and lack systematic analysis of key outcome indicators [11–13]. In clinical practice, the changes in pain patterns, dynamic changes in quality of life, and long-term tracking of growth and development in children with CP combined with PDS after ERCP treatment have not been clearly answered. These information gaps make it difficult for clinicians to balance the minimally invasive advantages and potential long-term risks when formulating long-term treatment strategies for pediatric CP combined with PDS, and there is a lack of evidence-based medicine to guide the individualized adjustment of treatment plans.

In view of this, this study aims to fill the above gaps. Through a retrospective cohort study, we systematically evaluated the long-term clinical outcomes of ERCP in the treatment of CP children. The study focuses on analyzing the long-term changes in pain patterns after ERCP treatment, the long-term improvement of growth indicators and quality of life (QoL), the incidence of long-term complications, and the long-term efficacy. The results of this study are expected to provide high-quality evidence-based support for optimizing the endoscopic treatment pathway of pediatric CP and formulating individualized long-term management plans, ultimately improving the long-term prognosis of affected children.

## 2. Materials and methods

### 2.1. Study design

All procedures were in accordance with the ethical standards of the institutional and/or national research committee and with the Helsinki Declaration of Helsinki. The informed consent was waived because the data were analyzed anonymously. The study received ethical approval from the Ethics Committee of West China Hospital, Sichuan University, located in Chengdu City, Sichuan Province, China, on May 21, 2025 (Approval Number: [NO.2025−996]). All methods employed in this study complied with relevant ethical guidelines and regulations and adhered to established standards for epidemiological observational studies. All data were strictly in compliance with the Declaration of Helsinki and relevant regulations on medical data privacy protection. All patients were de-identified and assigned unique identification numbers to ensure confidentiality. Since this study used anonymous data without patient intervention, informed consent was waived. We access this clinical data on May 25, 2025 for research purposes. This was a single-center retrospective cohort study. The subjects were children with CP combined with PDS who were hospitalized and treated in the Department of Pediatric Surgery, West China Hospital, Sichuan University, from January 2008 to December 2024.

Baseline and treatment data were obtained from the Hospital Information System (HIS) and the Picture Archiving and Communication Systems (PACS). The extracted content included basic patient information (gender, date of birth, height/weight at admission), time for CP diagnosis, details of pancreatic abdominal pain attacks, laboratory tests, imaging reports, and ERCP operation records.

A “active + passive” combined follow-up model was adopted. Active follow-up was conducted through standardized telephone questionnaires, and passive follow-up was conducted through the patients’ re-hospitalization for review (at least once a year, with review items including abdominal ultrasound/CT, growth measurement, blood glucose, and pancreatic enzyme level detection). The follow-up endpoint of this study was set as December 31, 2024. If a patient died during the follow-up period, the time of death was taken as the follow-up endpoint.

### 2.2. Study subjects

#### 2.2.1. Inclusion criteria.

(1) Age ≤ 18 years at the first diagnosis of CP, and the diagnosis of CP conforming to the 2002 Asia-Pacific Consensus Criteria for Chronic Pancreatitis [14]: Typical pancreatic abdominal pain; Imaging evidence; Abnormal pancreatic endocrine/exocrine function. The diagnosis was confirmed if at least 2 of the above criteria were met. (2) Diagnosis of PDS confirmed by at least one imaging examination. (3) Receipt of at least one ERCP treatment with complete ERCP operation records.

#### 2.2.2. Exclusion criteria.

(1) Diagnosis of pancreatic cancer within 2 years after CP confirmation. (2) Comorbidity with severe underlying diseases (e.g., severe congenital heart disease, decompensated liver cirrhosis, end-stage renal disease) that may affect growth and development or survival outcomes. (3) Pancreatic duct obstruction or stones caused by trauma, surgery, or other non-CP-related factors. (4) Follow-up data missing rate >30%.

### 2.3. Key definitions and diagnostic criteria

The etiology of chronic pancreatitis (CP) was classified using the TIGAR-O classification system [15].

#### 2.3.1. Classification of pancreatic abdominal pain [16].

Recurrent acute pancreatitis (RAP): ≥ 2 episodes of AP (serum amylase >3 times the upper limit of normal + typical abdominal pain), with no abnormal pancreatic imaging or function during the remission period.

Recurrent pain (RP): No AP changes on imaging, serum amylase <3 times the upper limit of normal, and abdominal pain attacks ≥1 time per month.

RAP + RP: Meeting the diagnostic criteria for both RAP and RP.

Chronic pancreatic pain (CPP): Abdominal pain lasting >8 hours per day or occurring ≥2 times per week for ≥2 months. Even if combined with AP or RP, it is classified into this category.

Visual Analog Scale (VAS): Ranging from 0 points (no pain) to 10 points (most severe pain), used for daily pain intensity assessment.

Izbicki Score: Including pain intensity (0–30 points), pain frequency (0–20 points), and impact on sleep/daily life (0–50 points), with a total score of 0–100 points, used for pain relief assessment at the follow-up endpoint.

Steatorrhea [17]: Clinical symptoms were persistent foul-smelling, greasy stools (≥3 bowel movements per day); Laboratory diagnosis: Detection of 3-day fecal fat excretion using the Van de Kamer method, with a result >14 g/day.

#### 2.3.2. Pancreatic Duct Stone (PDS) clearance rate.

Evaluated based on immediate post-operative pancreatic duct angiography:

Complete clearance: > 90% clearance of the volume of stones in the main pancreatic duct and its branches.

Partial clearance: 50%–90% clearance of the stone volume.

Failed clearance: < 50% clearance of the stone volume or no change in stone location.

#### 2.3.3. Post-ERCP complications.

Post-ERCP pancreatitis (PEP): Abdominal pain occurring within 24 hours after surgery, serum amylase >3 times the upper limit of normal, requiring medical treatment.

Bleeding: Hematemesis, melena, or hemoglobin decrease >20 g/L within 24 hours after surgery, requiring endoscopic hemostasis or blood transfusion.

Perforation: Occurrence of acute abdomen after surgery, with imaging confirming pancreatic duct or intestinal perforation.

#### 2.3.4. Recurrence of Pancreatic Duct Stones (PDS).

New stones detected by MRCP/CT re-examination after complete stone clearance, with an interval of ≥1 year from the last ERCP.

### 2.4. ERCP treatment process

All ERCP procedures in this study were performed by the same endoscopist with experience in pediatric ERCP. Fasting was required for 6–8 hours before surgery, and intravenous antibiotics were administered to prevent infection. A pediatric-specific duodenoscope was used to enter the descending part of the duodenum. After locating the Vater papilla, successful intubation into the main pancreatic duct was performed, followed by injection of contrast agent. Under X-ray fluoroscopy, the course of the main pancreatic duct, stone location, and stenosis were observed to clarify the scope of the lesion. Balloon lithotripsy or lithotomy basket was used for stone removal. For children with main pancreatic duct stenosis, a pediatric-specific plastic pancreatic duct stent was placed, and the length of the stent needed to cross the midline of the spine. The specific specifications were determined according to the diameter of the distal pancreatic duct and the degree of stenosis. For patients requiring temporary drainage after surgery, an endoscopic nasopancreatic drainage (ENPD) tube was placed for draining pancreatic juice and monitoring the condition.

### 2.5. Outcome indicators

Short-term outcomes included the post-ERCP pancreatic duct stone clearance rate and the incidence of post-ERCP complications (within 30 days after surgery). Long-term outcomes (follow-up ≥1 year) included:

(1) Pain-Related Outcomes

① Pain relief rate (complete relief: Izbicki score ≤10 points [18]; partial relief: Izbicki score >10 points but decreased by >50% compared with pre-treatment; no relief: failing to meet the criteria for partial relief); ② Changes in abdominal pain classification (changes in the proportion of RAP, RP, RAP + RP, and CPP before and after treatment); ③ Analgesic use rate.

(2) Growth Indicators

Changes in height, weight, and body mass index (BMI).

(3) Quality of Life Score

The PedsQL™4.0 Generic Core Scale was used to assess QoL, including 4 dimensions: physical function, emotional function, social function, and school function. Each item was scored from 1 to 5 points, which was converted to a 0–100 scale (higher scores indicate better QoL). The questionnaire was completed by the patients or their guardians.

(4) Other Outcomes

Recurrence rate of PDS; ② Incidence of new CP-related complications (pancreatic pseudocyst, pancreatic portal hypertension, new-onset diabetes, new-onset steatorrhea); ③ Subsequent surgery rate; ④ Pancreatic enzyme use.

### 2.6. Statistical analysis

Statistical analysis was performed using SPSS 22.0 (IBM Corp., USA) and R 4.2.1 software. All tests were two-tailed, and P < 0.05 was considered statistically significant. Normally distributed continuous variables were expressed as “mean ± standard deviation”, while non-normally distributed variables were expressed as “median [minimum, maximum] (M [Min, Max])”. The Shapiro-Wilk test was used for normality testing. Categorical data were expressed as “number of cases (percentage) [n (%)]”. For inter-group comparison of measurement data: the Student’s t-test was used for data with homogeneous variance, and the Welch’s t-test was used for data with heterogeneous variance; the Mann-Whitney U test was used for non-normally distributed variables. For categorical data: the χ² test was used when the theoretical frequency ≥5, and Fisher’s exact test was used when the theoretical frequency <5. S1 Table summarizing the statistical methods used for each outcome indicator.

Sample size estimation: This was a retrospective cohort study, and the sample size was determined via pre-determined estimation. Based on previous studies^11^ reporting that the complete pain relief rate of ERCP in the treatment of pediatric PDS was approximately 70%, with α = 0.05 (Type I error) and β = 0.2 (Type II error), and an allowable error of 10%, the minimum required sample size was estimated to be 68 cases using the formula. During the study period (January 2008 to December 2024), we screened all patients with CP and PDS who met the study’s inclusion/exclusion criteria at our center. A total of 90 eligible patients with complete clinical and follow-up data were ultimately enrolled, which exceeded the pre-estimated minimum sample size and further strengthened the reliability of the study results.

## 3. Results

### 3.1. Baseline information of patients

**Fig 1:**
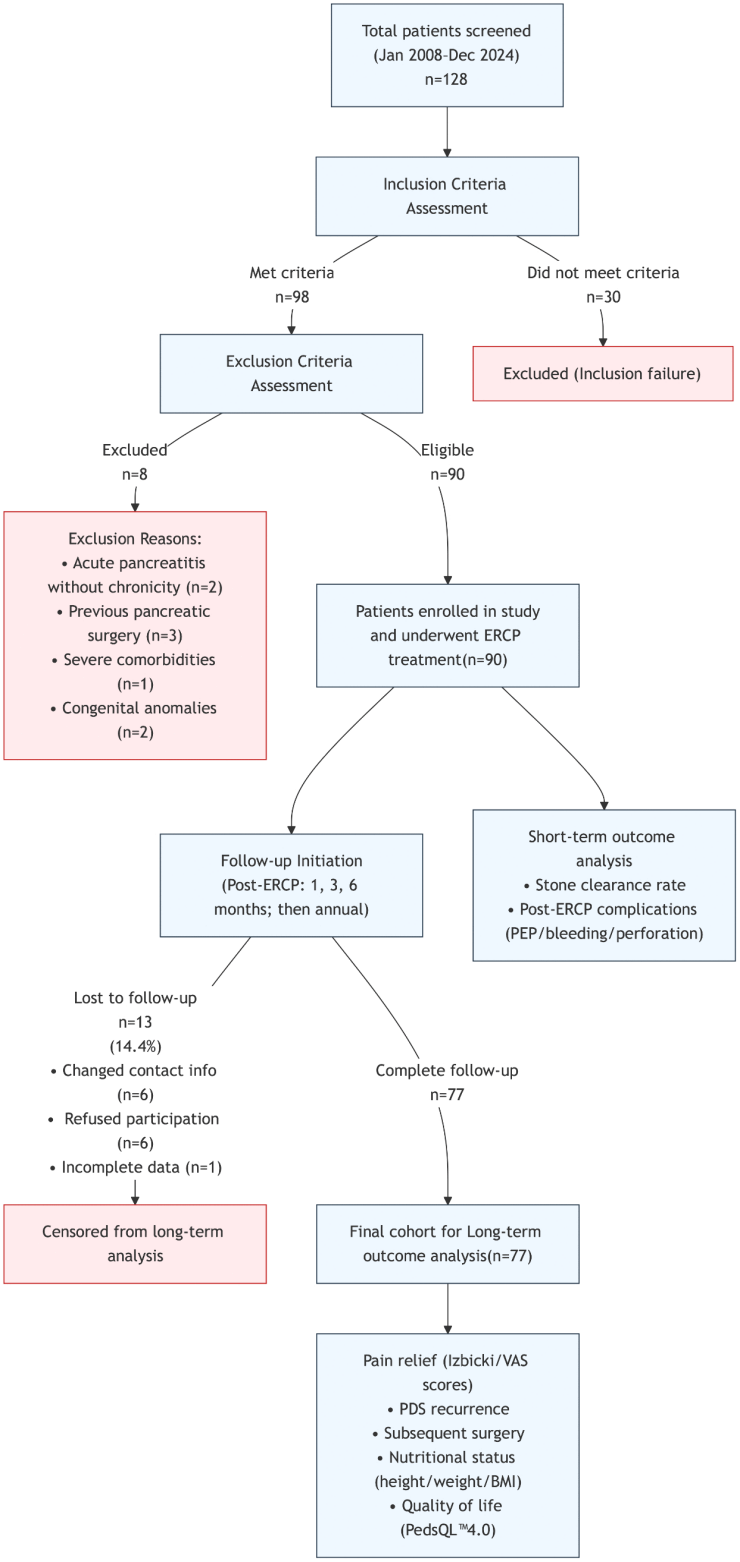
Study flow diagram of pediatric patients undergoing endoscopic retrograde cholangiopancreatography.

The complete patient journey from initial screening to final outcome analysis is illustrated in Study Flow Diagram (Fig 1). A total of 90 children with CP who received ERCP treatment in our hospital were finally included in this study. Their demographic and clinical baseline characteristics are shown in Table 1. In terms of demographic characteristics, there were slightly more male patients (51.1%, 46/90); the median age was 11.2 years, ranging from 4.40 to 16.7 years. In terms of clinical manifestations, abdominal pain was the most common initial symptom, accounting for 92.2% (83/90). The etiological spectrum was consistent with the characteristics of pediatric CP. According to the “TIGAR-O” classification system, idiopathic CP accounted for the highest proportion (78.9%, 71/90). The most common type of abdominal pain was recurrent pain (RP) (38.9%, 35/90). In terms of comorbidities, before the first ERCP treatment, 10.0% (9/90) of patients had been diagnosed with diabetes mellitus (DM), 4.4% (4/90) had steatorrhea, and the Charlson Comorbidity Index (CCI) showed that 82.2% (74/90) of patients had no obvious comorbidities (CCI = 0). In addition, family history analysis showed that 16.7% (15/90) of patients had relatives with DM, and 7.8% (7/90) had relatives with pancreatic diseases.

**Table 1:** Baseline characteristics of children with chronic pancreatitis prior to treatment.

Items	Female	Male	Overall	P value
	(N = 44)	(N = 46)	(N = 90)	
**Age**				
Median [Min, Max]	10.1 [4.40, 13.8]	11.7 [5.70, 16.7]	11.2 [4.40, 16.7]	0.052
**Initial manifestations**				
Abdominal pain	40 (90.9%)	43 (93.5%)	83 (92.2%)	
Endocrine/Exocrine dysfunction	2 (4.5%)	3 (6.5%)	5 (5.6%)	
Others	2 (4.5%)	0 (0%)	2 (2.2%)	0.322
**Aetiology**				
genetic	1 (2.3%)	1 (2.2%)	2 (2.2%)	
idiopathic	36 (81.8%)	35 (76.1%)	71 (78.9%)	
obstructive	6 (13.6%)	9 (19.6%)	15 (16.7%)	
toxic-metabolic	1 (2.3%)	1 (2.2%)	2 (2.2%)	0.903
**Steatorrhea**				
Absent	44 (100%)	42 (91.3%)	86 (95.6%)	
Present	0 (0%)	4 (8.7%)	4 (4.4%)	0.045
**Diabetes mellitus(DM)**				
Absent	39 (88.6%)	42 (91.3%)	81 (90.0%)	
Present	5 (11.4%)	4 (8.7%)	9 (10.0%)	0.673
**Biliary stricture**				
Absent	44 (100%)	45 (97.8%)	89 (98.9%)	
Present	0 (0%)	1 (2.2%)	1 (1.1%)	0.325
**Pancreatic pseudocyst**				
Absent	35 (79.5%)	39 (84.8%)	74 (82.2%)	
Present	9 (20.5%)	7 (15.2%)	16 (17.8%)	0.516
**Pancreatic sinistral portal hypertension**				
Absent	44 (100%)	45 (97.8%)	89 (98.9%)	
Present	0 (0%)	1 (2.2%)	1 (1.1%)	0.325
**Type of pain**				
CPP	3 (6.8%)	2 (4.3%)	5 (5.6%)	
RAP	11 (25.0%)	11 (23.9%)	22 (24.4%)	
RAP + RP	13 (29.5%)	15 (32.6%)	28 (31.1%)	
RP	17 (38.6%)	18 (39.1%)	35 (38.9%)	0.955
**Charlson Comorbidity Index**				
0	35 (79.5%)	39 (84.8%)	74 (82.2%)	
1_2	9 (20.5%)	6 (13.0%)	15 (16.7%)	
3_4	0 (0%)	1 (2.2%)	1 (1.1%)	
5+	0 (0%)	0 (0%)	0 (0%)	0.412
**Severe acute pancreatitis**				
Absent	44 (100%)	45 (97.8%)	89 (98.9%)	
Present	0 (0%)	1 (2.2%)	1 (1.1%)	0.325
**DM in relatives**				
Absent	37 (84.1%)	38 (82.6%)	75 (83.3%)	
Present	7 (15.9%)	8 (17.4%)	15 (16.7%)	0.85
**Pancreatic diseases in relatives**				
Absent	40 (90.9%)	43 (93.5%)	83 (92.2%)	
Present	4 (9.1%)	3 (6.5%)	7 (7.8%)	0.649

Abbreviations: CPP, Chronic pancreatic pain; RAP, Recurrent acute pancreatitis; RP, Recurrent pain; DM, diabetes mellitus.

### 3.2. Procedural characteristics of ERCP

A total of 104 ERCP treatments were performed in the 90 patients, including 77 cases (85.6%) receiving 1 treatment, 12 cases (13.3%) receiving 2 treatments, and 1 case (1.1%) receiving 3 treatments. The procedure-related characteristics are shown in Table 2. In terms of the location of pancreatic duct stones, stones in the pancreatic head accounted for the highest proportion (70.2%, 73/104), indicating that PDS in children mainly involves the pancreatic head. In terms of the morphology of the main pancreatic duct (MPD), simple pancreatic duct stones were the most common manifestation (55.8%, 58/104). In terms of endoscopic operation methods, all patients underwent pancreatic duct lithotomy (100%); among auxiliary operations, endoscopic retrograde pancreatic drainage (45.2%, 47/104) and endoscopic sphincterotomy (EST, 36.5%, 38/104) were the most widely used.

**Table 2:** Procedural characteristics of ERCP.

Items	Overall
	(N = 104)
**Location of stone**	
Body/tail	6 (5.8%)
Head	73 (70.2%)
Head and at least another location	25 (24.0%)
**Morphology of main pancreatic duct**	
Complex pathologic changes	27 (26.0%)
Indeterminant	8 (7.7%)
MPD stenosis and stone	11 (10.6%)
Pancreatic stone alone	58 (55.8%)
**Endoscopic treatment methods**	
Endoscopic sphincterotomy	38 (36.5%)
Endoscopic papillary balloon dilation	5 (4.8%)
Pancreatic duct stricture dilation	30 (28.8%)
Pancreatic duct lithotomy	104 (100%)
Endoscopic retrograde pancreatic drainage	47 (45.2%)
Endoscopic nasopancreatic drainage	36 (34.6%)
**Post ERCP complications**	
Bleeding	3 (2.9%)
PEP	7 (6.7%)
**Stone clearance**	
Complete	75 (72.1%)
Failed	10 (9.6%)
Partial	19 (18.3%)

Abbreviations: MPD, main pancreatic duct; PEP, post-ERCP pancreatitis.

Regarding post-operative complications and stone clearance efficacy: the overall incidence of post-ERCP complications was 9.6% (10/104), all of which were mild complications, including PEP in 6.7% (7/104) and post-operative bleeding in 2.9% (3/104). There were no perforations, infections, or other rare complications. All complications were cured after conservative medical treatment, with no conversion to surgery. In terms of stone clearance rate: the complete clearance rate was 72.1% (75/104), the partial clearance rate was 18.3% (19/104), and the clearance failure rate was 9.6% (10/104), indicating that ERCP has high clearance efficacy for pediatric PDS. The sub-analysis of stone location and ERCP efficacy show that complete stone clearance rate was significantly higher for pancreatic head stones (78.1%, 57/73) than for body/tail stones (50.0%, 3/6) or multi-location stones (64.0%, 16/25) (P = 0.032).

### 3.3. Long-Term Follow-Up

The follow-up endpoint of this study was December 2024. Among the 90 patients, 77 (85.6%) completed the full follow-up, with an average follow-up duration of 5.86 years (ranging from 2.18 to 13.89 years). Total 13 (14.4%) were lost to follow-up. The reasons for dropout are clarified as follows: (1) 6 patients (46.2%) had changed contact information without notification; (2) 6 patients (46.2%) refused further participation due to personal reasons; (3) 1 patient (7.7%) was excluded due to incomplete follow-up data (missing rate >30%, as per our exclusion criteria). The long-term follow-up outcomes are shown in Table 3.

**Table 3:** The long-term efficacy of ERCP for chronic pancreatitis.

Items	Pre-treatment	Post-treatment	P value
	(N = 77)	(N = 77)	
**Height**			
Median [Min, Max]	150 [112, 177]	154 [117, 184]	0.008
**Weight**			
Median [Min, Max]	35.0 [15.5, 69.0]	48.0 [28.5, 84.0]	0.001
**BMI(kg/m**^**2**^)			
Median [Min, Max]	15.6 [11.9, 24.4]	21.6 [16.9, 29.0]	0.001
**Quality of Life score**			
Median [Min, Max]	69 [56, 80]	85 [80, 90]	0.001
**Type of pain**			
No pain	0 (0%)	58 (75.3%)	
CPP	5 (6.5%)	1 (1.3%)	
RAP	19 (24.7%)	6 (7.8%)	
RAP + RP	25 (32.5%)	3 (3.9%)	
RP	28 (36.4%)	9 (11.7%)	0.001
**VAS**			
0	0 (0%)	45 (58.4%)	
1_3	16 (20.8%)	11 (14.3%)	
4_6	19 (24.7%)	16 (20.8%)	
7_10	42 (54.5%)	5 (6.5%)	0.001
**Usage of analgesics**			
Present	7 (9.1%)	3 (3.9%)	0.191
**Abdominal pain relief after ERCP**			
None	–	4 (5.2%)	
Partial relief	–	19 (24.7%)	
Completely relief	–	54 (70.1%)	
**Recurrence of PDS**	–	6 (7.8%)	
**Newly diagnosed DM**	–	4 (5.2%)	
**Newly diagnosed steatorrhea**	–	4 (5.2%)	
**Newly diagnosed PPC**	–	2 (2.6%)	
**Newly diagnosed PPH**	–	1 (1.3%)	
**Further surgery**	–	3 (3.9%)	
**Pancreatic Enzyme Administration**	–		
Not taking	–	10 (12.9%)	
Irregular taking	–	37 (48.1%)	
Regular taking	–	27 (35.1%)	
Unknown	–	3 (3.9%)	

Abbreviations: VAS, visual analogue scale; BMI, body mass index; PPC,pancreatic pseudocyst; PPH, pancreatic portal hypertension; PDS,pancreatic duct stones.

#### 3.3.1. Improvement of pain pattern and severity.

After ERCP treatment, both the abdominal pain pattern and severity of patients were significantly improved (P < 0.001). The overall complete pain relief rate was 70.1%, the partial relief rate was 24.7%, and only 5.2% had no relief. In terms of changes in abdominal pain classification: before treatment, “RAP+RP” (32.5%, 25/77) and “RP” (36.4%, 28/77) were the main types; after treatment, 75.3% (58/77) of patients achieved “no pain”. The proportion of each pathological type of abdominal pain decreased significantly: RAP decreased from 24.7% to 7.8%, RP decreased from 36.4% to 11.7%, RAP + RP decreased from 32.5% to 3.9%, and CPP decreased from 6.5% to 1.3%. The Visual Analog Scale (VAS) showed that before treatment, 54.5% of patients had a VAS score of 7–10 points (severe pain); after treatment, only 6.5% still had severe pain.

#### 3.3.2. Improvement of growth indicators.

After treatment, the height, weight, and body mass index (BMI) of patients were significantly increased compared with those before treatment (P < 0.01). The average height increased by 4 cm, the average weight increased by 13.0 kg (P = 0.001), and the median BMI increased from 15.6 kg/m² to 21.6 kg/m² (P = 0.001). Although BMI increased significantly, the BMI of both male and female patients was still lower than the median level of the same age group in the WHO Growth Standards for Children Aged 5–19 Years.

#### 3.3.3. Improvement of quality of life.

The PedsQL™4.0 Generic Core Scale was used to assess the quality of life. The median QoL score of patients increased significantly from 69 points (ranging from 56 to 80 points) before treatment to 85 points (ranging from 80 to 90 points) after treatment, with a statistically significant difference (P < 0.001).

#### 3.3.4. Long-term complications and subsequent treatment.

**Fig 2:**
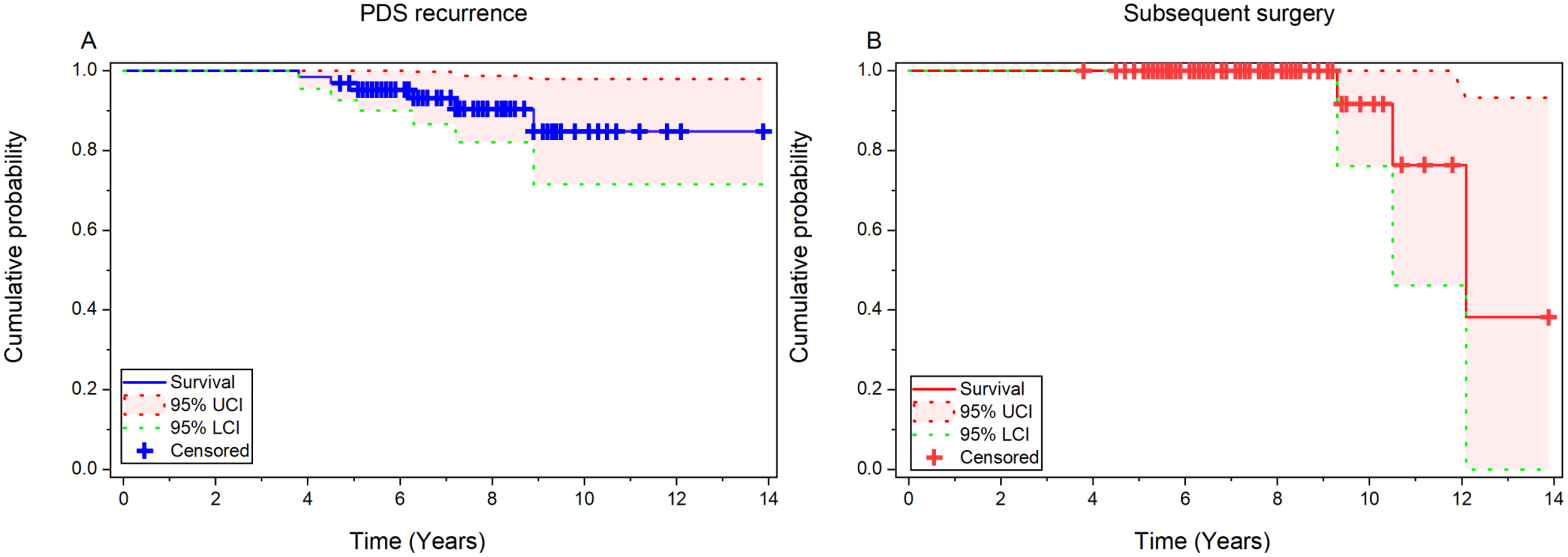
Kaplan-Meier curves for long-term outcomes after ERCP in children with CP and PDS. (A) Cumulative probability of PDS recurrence. A total of 6 PDS recurrence events occurred during follow-up. (B) Cumulative probability of subsequent surgery. Only 3 patients required subsequent surgical intervention.

No patient died during the follow-up period. Long-term complications were mainly mild dysfunction: 4 cases (5.2%) had new-onset DM, 4 cases (5.2%) had new-onset steatorrhea, 2 cases (2.6%) had new-onset pancreatic pseudocyst (PPC), and 1 case (1.3%) had new-onset pancreatic portal hypertension (PPH). All were controlled by conservative medical treatment, with no severe complications. A Kaplan-Meier curve is presented to visualize two key long-term outcomes (PDS recurrence and subsequent surgery) in 77 children with CP and PDS who completed follow-up after ERCP (Fig 2). Six cases (7.8%) had PDS recurrence, all of whom were patients with previous complete stone clearance. After recurrence, stone clearance was achieved through re-ERCP treatment. Only 3 patients underwent subsequent surgical treatment. During follow-up, the main pancreatic enzyme use pattern of patients was “irregular use” (48.1%, 37/77), and regular use accounted for 35.1% (27/77). Among the 9 pre-operative DM patients, 6 (66.7%) achieved stable blood glucose control with oral medications (no insulin required), 2 (22.2%) showed no change, and 1 (11.1%) had improved insulin sensitivity (reduced dosage). No patients experienced aggravation of DM. The 1 patient with pre-operative biliary stricture showed resolution of stenosis on follow-up imaging (MRCP) without further intervention. All 4 pre-operative steatorrhea patients reported symptom relief (fecal fat excretion <14 g/day by Van de Kamer method) after ERCP and pancreatic enzyme replacement therapy. Among the 16 pre-operative PPC patients, 14 (87.5%) resolved spontaneously, 2 (12.5%) required endoscopic drainage (no recurrence observed).

## 4. Discussion

This study systematically analyzed the clinical characteristics, procedural efficacy, and long-term follow-up outcomes of 90 children with CP who underwent ERCP. This is the first retrospective study with a long-term follow-up to evaluate the efficacy of ERCP for painful pancreatic stones in children with CP and to explore pain type conversion, quality of life, long-term complications and efficacy after treatment.

This study showed that children CP have distinct clinical characteristics, with idiopathic etiology being the main type, which is essentially different from the etiological distribution of adult CP. In this study, idiopathic CP accounted for 78.9%, which is consistent with the conclusions of multiple international studies. Lindley et al. pointed out that the etiology of pediatric CP differs from that of adults, with idiopathic and genetic types being the main types, and the interaction between environmental triggers and genetic susceptibility being the core mechanism [19]. The proportion of genetic CP in this study was only 2.2%, which is lower than that reported in some literatures [20]. This may be not all patients underwent genetic testing, leading to a certain degree of underdiagnosis of the etiology. In the future, whole-exome sequencing should be combined to further clarify the potential genetic background of idiopathic CP, which is consistent with the previous literature suggestion that multi-dimensional etiological screening is required for pediatric CP [6]. In addition, no gender difference was found in the etiological distribution of CP in this study, suggesting that the exploration of the etiology of pediatric CP does not need to focus on gender stratification, but rather on the potential mechanisms of idiopathic cases.

The complete pancreatic duct stone clearance rate of ERCP in this study was 72.1%, which is lower than the clearance rate of adult PDS (about 80%) [21]. However, considering that the pancreatic duct diameter of children is smaller and the operation space is limited, this result already reflects good technical feasibility. However, 9.6% of patients failed to achieve stone clearance, which may be attributed to several reasons. First, the pancreatic duct of children is slender, which limits the selection of instruments and increases the difficulty of stone removal [22]. Second, the location and distribution of stones may bring difficulties to stone removal. The pancreatic duct in the pancreatic tail has a steeper course and is far from the duodenoscope, making it difficult for instruments to reach. Third, chronic pancreatitis leads to reduced pancreatic juice secretion and increased viscosity, forming calcium carbonate in the form of calcite that adheres tightly to the pancreatic duct wall, making it difficult to clean, and some debris in the pancreatic duct branches and pancreatic parenchyma cannot be removed [23]. Therefore, these patients failed to achieve complete stone clearance. For patients with failed stone clearance, surgery is the only treatment option. A previous study on 626 children undergoing ERCP showed that the technical success rate was 96%, therapeutic ERCP accounted for 59%, and the complication rate was comparable to that of adults [24]. Tringali et al. pointed out that although the complication rate of therapeutic endoscopy in children is higher than that of diagnostic endoscopy, most complications are mild and controllable [22]. The sub-analysis of stone location and ERCP efficacy show that complete stone clearance rate was significantly higher for pancreatic head stones than for body/tail stonesor multi-location stones. Pancreatic head stones are more accessible to endoscopic instruments due to their proximity to the duodenum, whereas body/tail stones are located in a steeper and more distal segment of the pancreatic duct, limiting instrument maneuverability. This anatomical difference explains the higher clearance rate for head stones. ERCP is most effective for head-localized stones, supporting its potential role as first-line therapy for this subgroup. For tail/body stones, surgical intervention may be necessary when ERCP fails, citing anatomical limitations.

The incidence of post-ERCP complications in this study was 9.6% (all mild PEP or bleeding), which is consistent with the above literature, confirming that pediatric ERCP can be safely performed in experienced centers, especially for minimally invasive treatment of PDS. It is worth noting that in our study, stones in the pancreatic head accounted for 70.2%, and simple main pancreatic duct stones accounted for 55.8%. This anatomical feature provides a pathological basis for direct stone removal. It is consistent with the previous literature report that the pancreatic duct lesions in children with CP are mainly simple pancreatic duct dilatation or stones, and complex stenosis is rare [25], suggesting that ERCP can be preferentially selected as the first-line minimally invasive treatment for pediatric PDS, rather than direct surgical intervention. Post-ERCP pancreatitis (PEP) is one of the common complications after ERCP, a previous meta-analysis reported that the pooled incidence of PEP in children was 4% [26]. The incidence of PEP in our study was 6.7%, which is not significantly different from previous studies. PEP is caused by the combined effects of chemical, thermal, mechanical, hydrostatic, enzymatic, allergic, and microbial injuries caused by ERCP operation or pancreatic duct hydrostatic injury [27]. Therefore, it is necessary to standardize intraoperative operations, avoid repeated pancreatic duct intubation, use nasopancreatic drainage (ENPD) as needed, optimize intubation techniques to reduce the risk of PEP, and closely monitor after surgery, recheck serum amylase, and identify and treat PEP early with conservative measures [28].

A potential discrepancy between patients reporting “no pain” (75.3%) and those with a VAS score ≥1 (41.6%). The inconsistency is from different assessment scopes and clinical definitions of pain used by the two tools, each designed to capture distinct aspects of pain experience. First, the classification of “no pain” in this study was defined by the Izbicki Pain Score (≤10 points), a validated comprehensive tool that evaluates three interrelated dimensions of pain: intensity, frequency, and impact on daily activities/sleep. A score ≤10 indicates not just the absence of severe pain, but the remission of clinically meaningful pain. In contrast, the VAS (0–10 points) is a single-item tool that measures instantaneous pain intensity at the time of assessment, without accounting for frequency or functional impact. This distinction is particularly relevant in pediatric patients, where subjective perception of discomfort varies widely: some children may describe mild, fleeting sensations as a VAS score ≥1, even if these sensations do not interfere with their daily lives or meet the criteria for pathological pain. Notably, this discrepancy underscores the value of using multi-modal pain assessment in pediatric CP research. Relying solely on VAS might overestimate pain burden by capturing trivial discomfort, while exclusive use of the Izbicki score could overlook subtle changes in pain intensity. By combining both tools, we balanced sensitivity to mild symptoms with a focus on clinically relevant outcomes, ensuring that our result reflects meaningful improvements in patient well-being, rather than superficial changes in subjective discomfort reporting. The complete pain relief rate after ERCP in our study was 70.1%, while the 10-year overall pain relief rate after total pancreatectomy with islet cell auto-transplantation (TPIAT) in children was 77.1% [29]. The two rates are similar, suggesting that for children suitable for endoscopic intervention, ERCP can achieve long-term pain relief effects similar to those of surgery (TPIAT). Notably, TPIAT is indicated for end-stage refractory CP, whereas ERCP is a first-line intervention for PDS in children with early-to-moderate CP. These two treatments target different patient populations, and thus direct comparison should be interpreted with caution. ERCP’s advantage lies in its minimally invasive nature, which avoids the surgical trauma and long-term complications associated with TPIAT, making it a preferred option for eligible pediatric patients. Long-term follow-up in this study showed that the proportion of all types of abdominal pain (RAP, RP, RAP + RP, CPP) decreased significantly after ERCP. This result not only provides direct clinical evidence for the effectiveness of ERCP in pain management related to pediatric pancreatic duct stones but also fills the evidence gap in the field of minimally invasive treatment of pediatric pancreatic duct stones from the perspective of long-term efficacy, which has important guiding value for the clinical practice of pediatric pancreaticobiliary diseases.

The core pathophysiological basis of abdominal pain caused by pancreatic duct stones in children lies in the vicious cycle of increased intrapancreatic duct pressure and persistent pancreatic inflammatory response caused by pancreatic duct obstruction [30]. The significant decrease in the proportion of abdominal pain after ERCP in our study can be explained by the following three mechanisms. First, ERCP directly relieves pancreatic duct obstruction through endoscopic stone removal, reduces intrapancreatic duct pressure, and restores the pancreatic juice drainage channel. Previous studies have confirmed that ERCP has a clear drainage effect on pediatric pancreaticobiliary obstructive diseases (such as common bile duct dilatation and pancreaticobiliary malformations). For example, a study on 58 children with common bile duct dilatation and stones showed that the bile duct diameter significantly decreased and liver function indicators improved after ERCP, confirming the effectiveness of endoscopic relief of obstruction [31]. This relief of obstruction can quickly reduce intrapancreatic duct pressure and reduce pain stimulation from the source, which is the key reason for the decreased frequency of RAP and RP episodes. Second, ERCP improves the pathological basis of CPP. Long-term pancreatic duct obstruction leads to chronic inflammation and fibrosis of pancreatic tissue, while the restoration of pancreatic juice drainage after ERCP can break this pathological process. A study on Indian children found that the inflammatory indicators of patients with chronic pancreatitis significantly decreased and clinical symptoms were relieved after ERCP [32]. Similarly, the decrease in the proportion of CPP in our study may be related to the reduction of chronic pancreatic inflammation after ERCP, which improves the long-term symptoms of CPP. Third, the recurrent episodes of RAP and RP can increase the pain sensitivity of children, while ERCP can break this cycle. For example, Qin et al. found that the BMI of children significantly increased after ERCP, suggesting that pain relief may improve the eating and nutritional status of children, further indirectly promoting the recovery of pancreatic function and forming a positive feedback on efficacy [33].

Previous studies on pediatric ERCP mostly focused on diseases such as gallstone pancreatitis and pancreaticobiliary malformations, and although they confirmed the safety and short-term efficacy of ERCP [34], long-term follow-up studies targeting the specific population of pancreatic duct stones and covering all types of pain (CPP, RAP and RP) are still scarce. This study is the first to systematically confirm the long-term improvement effect of ERCP on multiple types of pain related to pediatric pancreatic duct stones. Most previous studies on pediatric ERCP used stone clearance rate and post-operative complication rate as the main endpoints. For example, a multi-center study compared the efficacy of ERCP and the surgery-first strategy in pediatric common bile duct stones, and although it mentioned the short-term symptom relief effect of ERCP, it did not conduct an in-depth analysis of the differences in pain types [35]. Through long-term follow-up, our study found that ERCP significantly improves CPP, RAP, and RP, among which the long-term relief of CPP is particularly important. As a type of chronic pain, CPP is much more difficult to treat than acute pain. The results of our study provide long-term evidence for ERCP in improving chronic pancreatic pain in children for the first time, making up for the shortcomings of existing studies. In addition, our study highlights the advantages of ERCP in the treatment of pediatric pancreatic duct stones. For pediatric PDS, traditional treatment methods such as surgical pancreatic ductotomy and stone removal can clear stones, but they are associated with large surgical trauma, long post-operative recovery time, and may affect the pancreatic development of children [36]. The significant improvement in pain after ERCP in our study further supports its role as a minimally invasive treatment option for PDS children with pain, especially for children who cannot tolerate surgery or hope to reduce trauma. The recurrence of pain related to pediatric PDS is insidious, and short-term follow-up may miss pain events with delayed onset. Our study adopted a long-term follow-up design, which can more truly reflect the long-term efficacy of ERCP and avoid the misjudgment of temporary pain relief in short-term studies, resulting in more reliable results. The CPP, RAP, and RP often coexist or transform into each other in children with PDS. Previous studies mostly focused on a single type of pain, making it difficult to comprehensively evaluate the treatment effect. This study simultaneously analyzed the changes in the three types of pain, and the results are more in line with the clinical needs of comprehensive pain management, providing more comprehensive references for clinical decision-making.

The relief of abdominal pain in patients after ERCP treatment has greatly improved their quality of life and is also conducive to improving their growth and development. In our study, after ERCP treatment, the quality of life score of CP children significantly increased from 69 points before treatment to 85 points after treatment. This result reflects that ERCP treatment not only relieves symptoms but also effectively reverses the multi-dimensional damage of CP to quality of life. Before ERCP treatment, children often had a decreased quality of life due to recurrent abdominal pain. After ERCP, 70.1% of children achieved complete pain relief, and the proportion of severe pain decreased from 54.5% to 6.5%, which directly promoted the improvement of physical function scores. This is completely consistent with the conclusion proposed by Perito et al. that pain relief is the core prerequisite for improving the quality of life of children with CP [37]. In our study, the BMI of children significantly increased after ERCP, but their BMI was still lower than the standard BMI. The possible reasons are as follows: First, the median age of children with CP undergoing ERCP was 11.2 years, which is a critical period for children’s growth and development. Insufficient intake and absorption of nutrients will affect physical condition. Second, domestic and foreign guidelines suggest that pancreatic enzyme replacement therapy should be administered to children with pancreatic exocrine insufficiency, growth retardation, and malnutrition [38]. However, in our study, only 35.1% of patients took pancreatic enzymes regularly, and the dosage of pancreatic enzymes was insufficient, which affected the digestion and absorption of fat, carbohydrates, and proteins in patients and led to the persistence of malnutrition. Third, some patients consciously reduced food intake, especially fat intake, due to excessive worry about abdominal pain attacks, which is also not conducive to the growth and development of children. The occurrence of DM and steatorrhea is a sign of decreased pancreatic endocrine and exocrine functions. During the follow-up period of our study, the incidence of DM and steatorrhea in children with CP was 5.2%. This result is similar to that of foreign studies. Among the 9 pre-operative DM patients, no patients experienced aggravation of DM. The 1 patient with pre-operative biliary stricture showed resolution of stenosis. All 4 pre-operative steatorrhea patients reported symptom relief after ERCP and pancreatic enzyme replacement therapy. Among the 16 pre-operative PPC patients, 14 resolved spontaneously, 2 required endoscopic drainage. These results confirm that ERCP not only prevents new complications but also alleviates pre-existing conditions. The progression rate of pancreatic endocrine and exocrine insufficiency in patients with early-onset CP is slower than that in patients with late-onset CP [39]. However, previous studies have shown that although children with CP have a lower risk of pancreatic endocrine and exocrine insufficiency in the early stage of the disease, the risk gradually increases after long-term follow-up. The cumulative incidence of steatorrhea in the pediatric group 30 years after the onset of CP is similar to or even higher than that in adults [40]. Pancreatic enzyme use remains high because pancreatic enzyme replacement therapy (PERT) targets exocrine insufficiency rather than pain. Even with pain remission, patients may retain mild-to-moderate pancreatic exocrine insufficiency due to pancreatic fibrosis, requiring continued PERT to optimize nutrient absorption and growth.

Of course, this study also has certain limitations. First, this was a single-center retrospective cohort study, which may have selection bias. Only 2.2% of patients underwent genetic testing, leading to potential underdiagnosis of hereditary CP. Second, this study did not compare ERCP with other treatment methods such as extracorporeal shock wave lithotripsy and surgical operations, so the relative advantages of ERCP cannot be clarified. Finally, the quality of life and other indicators in this study relied on retrospective questionnaires, which may have recall bias, and prospective recording is needed to improve accuracy. The follow-up period covered the critical transition from childhood to early adulthood, and further extension may not add significant value given the stable long-term outcomes observed. However, long-term monitoring of adult-onset complications remains warranted in future studies.

## 5. Conclusion

This study confirms that ERCP is effective minimally invasive treatments for pancreatic stones in children with CP, significantly improving long-term pain, growth and development, and quality of life. Clinically, attention should be paid to long-term post-operative management, and prospective comparative studies should be conducted to further optimize the treatment plan, ultimately improving the prognosis of children with CP and PDS.

## Supporting information

S1 Table Summary of Statistical Methods for Each Outcome Indicator. (DOCX)
