# Supplementary material for: Long-term clinical outcomes of endoscopic retrograde cholangiopancreatography for pancreatic duct stone treatment in children with chronic pancreatitis
Source: PLoS One. 2026 Feb 27;21(2):e0336638. doi: 10.1371/journal.pone.0336638 (PMC12948049; doi:10.1371/journal.pone.0336638)
Supplement: S1 Table — (DOCX) [file pone.0336638.s001.docx]

**Supplementary Table S1: Summary of Statistical Methods for Each Outcome Indicator**

| Outcome Indicator | Data Type | Statistical Method | Reference to Results Section |
| --- | --- | --- | --- |
| **Short-term Outcomes** |  |  |  |
| 1. Pancreatic duct stone clearance rate (Complete/Partial/Failed) | Categorical | χ² test (if theoretical frequency ≥ 5); Fisher’s exact test (if theoretical frequency < 5) | Section 3.2 |
| 2. Incidence of post-ERCP complications (PEP/bleeding/perforation) | Categorical | χ² test (if theoretical frequency ≥ 5); Fisher’s exact test (if theoretical frequency < 5) | Section 3.2 |
| **Long-term Outcomes** |  |  |  |
| 3. Pain relief rate (Complete/Partial/No relief) | Categorical | χ² test (if theoretical frequency ≥ 5); Fisher’s exact test (if theoretical frequency < 5) | Section 3.3.1 |
| 4. Change in abdominal pain type (RAP/RP/RAP+RP/CPP/No pain) | Categorical | χ² test (if theoretical frequency ≥ 5); Fisher’s exact test (if theoretical frequency < 5) | Section 3.3.1 |
| 5. Visual Analog Scale (VAS) score (pre- vs. post-treatment) | Continuous | Shapiro-Wilk test for normality; <br> - If normal: Paired Student’s t-test; <br> - If non-normal: Wilcoxon signed-rank test | Section 3.3.1 |
| 6. Height (pre- vs. post-treatment) | Continuous | Shapiro-Wilk test for normality; <br> - If normal: Paired Student’s t-test; <br> - If non-normal: Wilcoxon signed-rank test | Section 3.3.2 |
| 7. Weight (pre- vs. post-treatment) | Continuous | Shapiro-Wilk test for normality; <br> - If normal: Paired Student’s t-test; <br> - If non-normal: Wilcoxon signed-rank test | Section 3.3.2 |
| 8. Body Mass Index (BMI) (pre- vs. post-treatment) | Continuous | Shapiro-Wilk test for normality; <br> - If normal: Paired Student’s t-test; <br> - If non-normal: Wilcoxon signed-rank test | Section 3.3.2 |
| 9. PedsQL™4.0 Generic Core Scale score (pre- vs. post-treatment) | Continuous | Shapiro-Wilk test for normality; <br> - If normal: Paired Student’s t-test; <br> - If non-normal: Wilcoxon signed-rank test | Section 3.3.3 |
| 10. Recurrence rate of pancreatic duct stones (PDS) | Categorical | χ² test (if theoretical frequency ≥ 5); Fisher’s exact test (if theoretical frequency < 5) | Section 3.3.4 |
| 11. Incidence of newly diagnosed diabetes mellitus (DM) | Categorical | χ² test (if theoretical frequency ≥ 5); Fisher’s exact test (if theoretical frequency < 5) | Section 3.3.4 |
| 12. Incidence of newly diagnosed steatorrhea | Categorical | χ² test (if theoretical frequency ≥ 5); Fisher’s exact test (if theoretical frequency < 5) | Section 3.3.4 |
| 13. Rate of subsequent surgery | Categorical | χ² test (if theoretical frequency ≥ 5); Fisher’s exact test (if theoretical frequency < 5) | Section 3.3.4 |
| 14. Pancreatic enzyme use pattern (Regular/Irregular/Not taking) | Categorical | χ² test (if theoretical frequency ≥ 5); Fisher’s exact test (if theoretical frequency < 5) | Section 3.3.4 |

**Notes:**

All statistical tests were two-sided; *P* < 0.05 was considered statistically significant.

Abbreviations: ERCP = endoscopic retrograde cholangiopancreatography; PEP = post-ERCP pancreatitis; RAP = recurrent acute pancreatitis; RP = recurrent pain; CPP = chronic pancreatic pain; BMI = body mass index; PDS = pancreatic duct stones.
